# Microbial Translocation Contribute to Febrile Episodes in Adults with Chemotherapy-Induced Neutropenia

**DOI:** 10.1371/journal.pone.0068056

**Published:** 2013-07-16

**Authors:** Michelle Wong, Babilonia Barqasho, Lars Öhrmalm, Thomas Tolfvenstam, Piotr Nowak

**Affiliations:** 1 Department of Medicine, Solna, Infectious Disease Unit, Center for Molecular Medicine, Karolinska University Hospital, Stockholm, Sweden; 2 Department of Laboratory medicine, Division of Microbiology, Karolinska Institutet, Huddinge, Sweden; 3 Department of Medicine, Huddinge, Infectious Disease Unit, Karolinska Institutet, Karolinska University Hospital, Huddinge, Sweden; University of Malaya, Malaysia

## Abstract

In this study we sought to determine the contribution of microbial translocation to febrile episodes with no attributable microbiological cause (Fever of Unknown Origin, FUO) in an adult febrile neutropaenic cohort. Endotoxin concentrations were measured with the chromogenic Limulus Amoebocyte Assay and used as a direct measure of bacterial products whilst soluble CD14 (sCD14), measured with ELISA was selected as an indicator of the early host response to endotoxins. Endotoxin concentrations in this cohort were generally elevated but did not differ with the presentation of fever. Further stratification of the febrile episodes based on the microbiological findings revealed significantly (p = 0.0077) elevated endotoxin concentrations in FUO episodes compared with episodes with documented bacterial and viral findings. sCD14 concentrations were however, elevated in febrile episodes (p = 0.0066) and no association was observed between sCD14 concentration and microbiological findings. However, FUO episodes and episodes with Gram-negative bacteraemia were associated with higher median sCD14 concentrations than episodes with Gram-positive bacteraemia (p = 0.030). In conclusion, our findings suggest that in the absence of microbiological findings, microbial translocation could contribute to febrile episodes in an adult neutropaenic cohort. We further observed an association between prophylactic antibiotic use and increased plasma endotoxin concentrations (p = 0.0212).

## Introduction

Neutropenia is a common complication in patients undergoing chemotherapy for malignancies and is one of the most important risk factors for infections. Infections account for substantial morbidity and mortality and fever is often the only indication of infection in this patient group. [1] Empirically, prompt administration of broad-spectrum antimicrobials has been one of the important contributions to increased event free survival and overall survival among these patients. [2] Today, bacteraemia is identified in 23%–33% [3], [4], [5], [6], [7] of all patients, and studies of the viral contribution to the aetiological panorama are still limited but estimated to be 30% [8]. This leaves, approximately a third of febrile episodes unexplained despite extensive microbiological investigations and these are thus termed fever of unknown origin (FUO). [9], [10].

Cytotoxic agents administered as treatment for the underlying malignancies often cause intestinal mucosal damage and subsequent inflammation- mucosistis. This, process disrupts the balance between the normal bowel bacterial flora and host immune system, creating a milieu where microbial products could translocate into systemic circulation. Endotoxins, more specifically, lipopolysaccharides (LPS) are found commonly on the outer membrane of Gram negative bacteria. Their presence in the peripheral circulatory system could thus signify potential bacterial invasion, requiring rapid host response. Potent pyrogens, endotoxins activate the inflammatory host defence via binding to soluble SC14 (sCD14) which, in turn initiates downstream cytokines important for the clearance of bacterial infections. Elevated endotoxins had been reported in studies with immunocompromised cohorts, for example, HIV-1 [11] and patients with haematological malignancies and is used as a measure of microbial translocation. [12], [13], [14], [15].

Since the manifestation of febrile state is dependent upon the pro-inflammatory response towards endotoxins, sCD14, a receptor for LPS could be a suitable immediate early response biomarker for fevers associated with microbial translocation.

In this study we thus sought to determine the contribution of microbial translocation to FUO episodes in an adult febrile neutropaenic cohort using endotoxin and sCD14 concentrations in the plasma as markers for microbial translocation.

## Materials and Methods

### Study Population

During a 26 month period (January 2008 to February 2010), adult patients with haematological disorders at the Karolinska University Hospital, Stockholm were, after informed consent, included in a cross sectional study where the inclusion criterion was chemotherapy-induced neutropenia (absolute neutrophil count ≤500/mm^3^). Patients that developed fever (auricular temperature >38.0°C twice within an hour or ≥38.5°C at one occasion) were sampled within 72 hours from fever onset, whereas patients without fever were sampled upon routine medical appointments during the neutropenia episode. Whole blood was collected in EDTA-tubes and plasma was prepared by centrifugation before storage at −80°C until use. Additional blood and nasal pharyngeal aspirates (NPA) samples were collected for microbiological testing. Medical records were retrospectively acquired for all included patients. The study was approved by The Regional Ethical Review Board in Stockholm, permit numbers 2007/1213-31/4 and 2008/1300-32.

### Microbiological Analyses

All patients were screened with the same diagnostic panel. Blood bacterial cultures and PCR viral diagnostics for adenovirus, Epstein - Barr virus and cytomegalovirus were performed, according to clinical routine, by the local clinical microbiology laboratory, Karolinska University Hospital. Additionally, viral diagnostics for BK polyomavirus from blood samples and, respiratory viruses (Human rhinovirus, respiratory syncytial virus, coronavirus, influenza virus A and B, parainfluenza virus 1–4, metapneumovirus, parvovirus B19, KI/WU polyoma virus and adenovirus) from NPA samples were performed in-house and based on PCR methods described in previous studies [16], [17], [18], [19], [20], [21], [22] and Ohrmalm et al. [23].

### Chromogenic Limulus Amoebocyte Lysate (LAL) Assay with Diazo Coupling

LPS was detected in patient plasma by the endpoint chromogenic Limulus Amebocyte Lysate (LAL) method using the QCL-1000 kit (Lonza group ltd., Switzerland) with modifications in the visualisation of the cleaved substrate. [24] All reagents and consumables were endotoxin –free (Lonza).

Briefly, plasma was diluted 1∶10 with 10 mM MgCl_2_ solution and heat inactivated at 80°C for 12 minutes. The products were then cooled at room temperature for 15 minutes and 50 µl of the sample was added to empty wells of an endotoxin free flat bottomed 96-well plate (Lonza). The background absorbance was measured at 540 nm (Labsystems Multiskan MCC 340, Deltasoft Multiskan III 2.26) and the plate was placed in a 37°C block heater to start the microplate assay (QCL-1000). Thereafter, 50 µl of pre-warmed (37°C) LAL lysate (Lonza) was added to each well and incubated for 10 min followed by 100 µl of pre warmed 37°C Chromogenic Substrate Solution (Lonza) and incubated for a further 6 minutes. The reaction was stopped by addition of 50 µl of sodium nitrate (0.3 mg/mL) in 0.48 N HCl to each well. Subsequently, 50 µl of ammonium sulphamate (2.25 mg/mL) dissolved in water was added to each well, followed by 50 µl of N.E.D.A [N-(1-naphthyl) ethylene-diamine dihydrochloride, 0.525 mg/mL], producing a brilliant magenta-colored derivative. The color change was measured at 540 nm within 15 minutes. Endotoxin standards (1 EU/mL to 0.0625 EU/mL) prepared in endotoxin free water were analysed alongside the samples as were, LPS spiked samples as controls for assay inhibition. To calculate the LPS levels, the final absorbance reading was subtracted from the background reading and derived from a linear regression of the absorbance readings of the known endotoxin standard concentrations.

### sCD14 Measurements

Plasma concentrations of sCD14 were measured with a commercially available, Quantikine ® Human sCD14 immunoassay kit (R&D systems, Inc., Minneapolis, USA).

### Peripheral Blood Cell Populations

Absolute neutrophil and leucocyte counts were obtained retrospectively from medical records of routine blood analyses. In addition, the absolute count of lymphocytes, CD4+ T cells, CD8+ T cells, CD 19+B cells, CD56+NK cells, and monocytes was determined in 59 episodes where the material was available, by flow cytometry using the BD Multitest antibodies and TruCount tubes (BD Biosciences, NJ, USA).

### Statistical Analysis

Statistical analyses were made with the Prism suite (Graphpad Inc., CA, USA). Fisher’s exact test and Mann-Whitney test were used for investigating the patients’ general characteristics. Mann-Whitney test was further used for analyses involving continuous data for two groups and Kruskal-Wallis test was used for all other analyses where comparison of continuous data for 3 groups or more were required. Correlations were calculated by using Spearman’s rank test. A p-value <0.05 was considered statistically significant.

## Results

A total of 103 febrile neutropaenic episodes and 42 afebrile neutropaenic episodes were included in the study (Table 1). The majority of patients were treated for acute leukaemias and none were diagnosed with invasive fungal infections. Microbiological findings were assumed, for the purpose of this study, to be the primary cause of fever and episodes with no microbiological findings are classified as ‘fever with unknown origin’ (FUO).

**Table 1:** Characteristics and laboratory data on patients with febrile neutropenia and neutropenic patients without fever.

Characteristics	Fever (n = 103)	Without fever (n = 42)	p-value
No. of females (%)	41 (40)	17 (40)	n.s.
Age [years], median (range)	58 (20–86)	62 (28–85)	n.s.
Underlying disease (%)			
Acute leukemia/MDS	53 (51)	22 (52)	n.s.
Chronic myeloid leukemia	0 (0)	1 (2)	n.s.
Chronic lymphocytic leukemia	2 (2)	4 (10)	n.s.
Non-Hodgkin lymphoma	28 (27)	11 (26)	n.s.
Myeloma	14 (14)	4 (10)	n.s.
Hodgkin’s disease	3 (3)	0 (0)	n.s.
Others	3 (3)	0 (0)	n.s.
Cell count^a^, median			
Neutrophils,[10^9^ cells/L]	<0.01 (<0.01–<0.01)	0.2 (<0.01–0.3)	<0.0001
Lymphocytes, [10^8^ cells/L]	1.07 (0.42–2.17)	4.95 (1.81–9.76)	<0.0001
Monocytes, [10^7^ cells/L]	4.48 (0.10–16.39)	7.29 (1.69–17.97)	n.s.
Microbiological findings (%)			
Bacteria	37 (36)	Not done	-
Virus	28 (27)	5 (12)	n.s.

a Absolute neutrophile counts were obtained from medical records whilst cell counts for lymphocytes and monocytes were performed in 59 episodes where the material was available.

### Endotoxin

**Figure 1:**
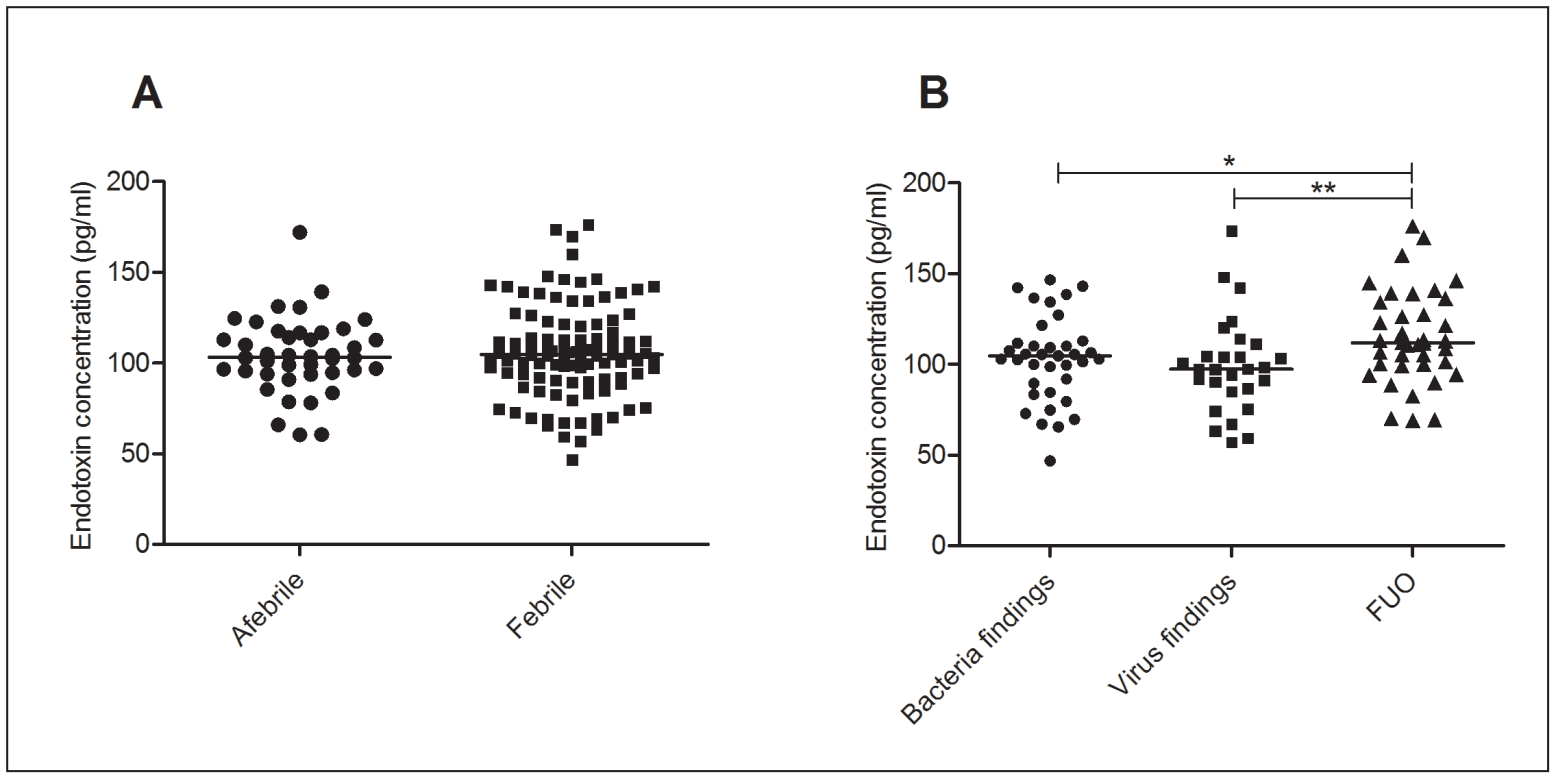
Endotoxin concentrations stratified based on febrile status and microbiological findings. Endotoxin concentrations measured in febrile (n = 103) and afebrile (n = 42) episodes (A). Endotoxin concentrations in febrile neutropaenic episodes with bacteria (n = 37), virus (n = 28) and FUO (n = 38) (B). * p<0.05, *** p<0.0001.

In this neutropaenic cohort, endotoxin concentrations were not different between the febrile and afebrile episodes (Figure 1a). Febrile patients (median = 104.7 pg/mL [IQR = 91.3 pg/mL–121.4 pg/mL]) had similar plasma endotoxin concentrations as the afebrile patients (median = 103.0 pg/mL [94.5 pg/mL–116.6 pg/mL]). Further stratification of the febrile episodes based on the microbiological findings revealed significantly (p = 0.0077) elevated endotoxin concentrations in FUO episodes (median = 111.8 pg/mL [IQR = 99.8 pg/mL–134.9 pg/mL]) whilst episodes with documented bacterial (104.7 pg/mL [IQR = 87.1 pg/mL–112.3 pg/mL]) and viral (97.3 pg/mL [IQR = 85.45 pg/mL–109.4 pg/mL]) findings were similar (Figure. 1b). No correlation was observed between viral findings and endotoxin concentrations.

**Figure 2:**
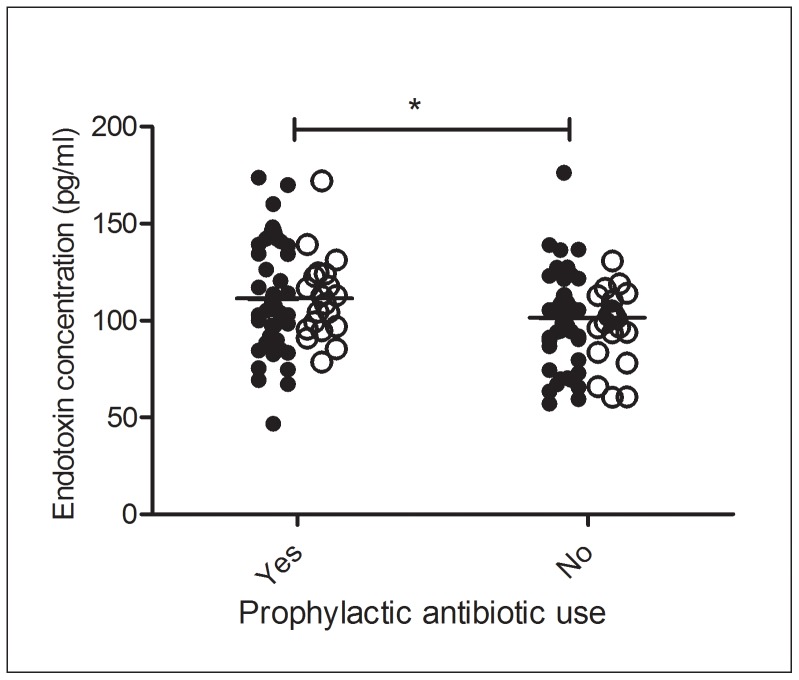
Endotoxin concentrations with (n = 68) and without (n = 77) prophylactic antibiotics use. Filled circles indicate febrile episodes and open circles indicate afebrile episodes. *p<0.05.

Interestingly, use of prophylactic antibiotics (ciprofloxacin and combination therapy with trimetoprim and sulfamethoxazole) was correlated with higher (p = 0.0212) endotoxin levels at a median of 110.9 pg/mL (IQR = 93.2 pg/mL–139.1 pg/mL) compared to a median of 101.4 pg/mL (IQR = 90.4 pg/mL–110.9 pg/mL) in episodes where prophylactic antibiotics were not administered (Figure 2). No association was seen between endotoxin concentration and neither other forms of antibiotics (administered before sampling), the underlying disease nor the general treatment regime.

### sCD14

**Figure 3:**
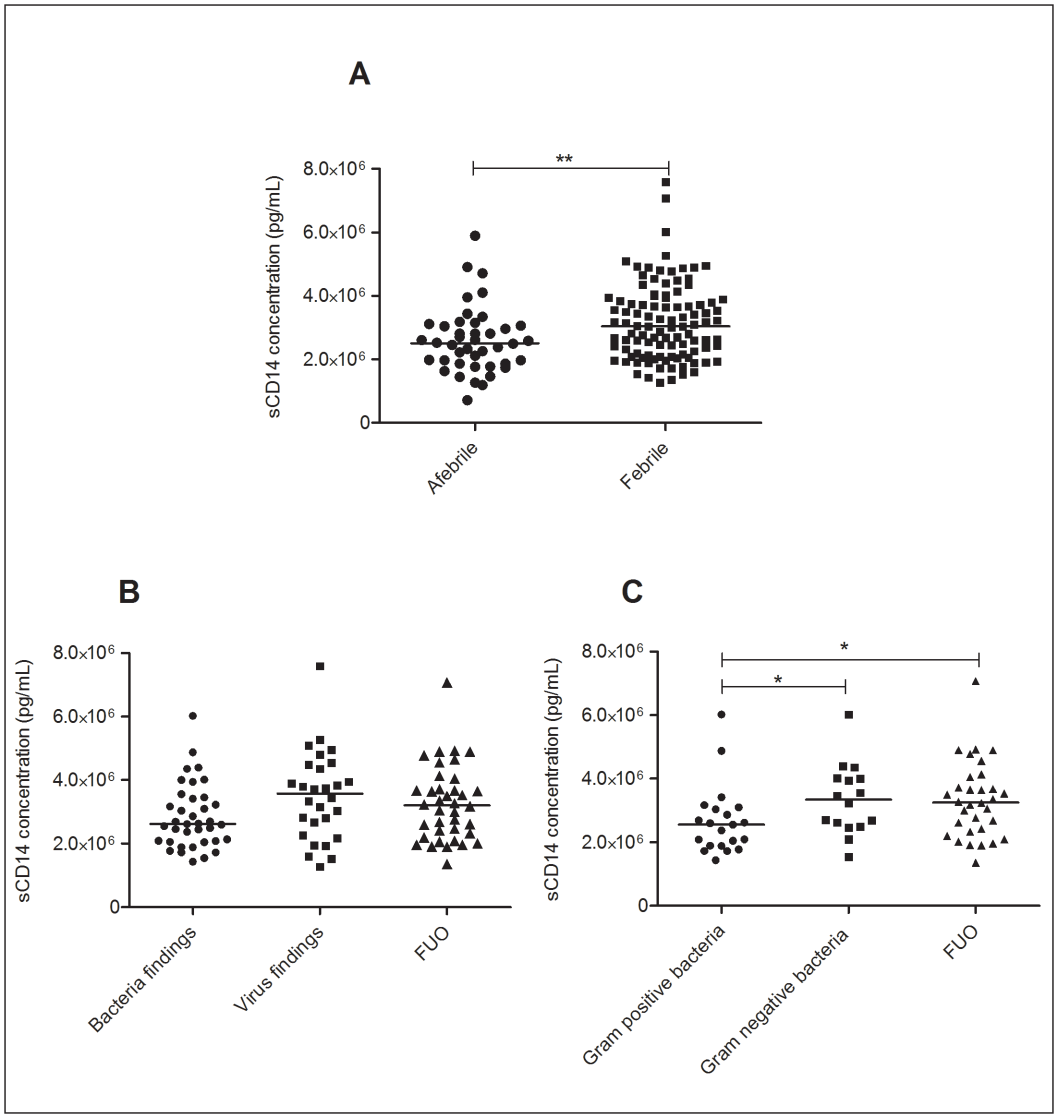
sCD14 concentrations stratified according to microbiological findings. sCD14 concentrations measured in febrile (n = 103) and afebrile (n = 42) episodes (A). sCD14 concentrations in febrile neutropaenic episodes with bacteria (n = 37), virus (n = 28) and FUO (n = 38) (B). sCD14 concentrations measured in episodes with Gram-positive (n = 21) and Gram-negative (n = 16) bacteria findings (C). * p<0.05, ** p<0.001.

A positive association was observed between sCD14 concentrations and manifestation of febrile status (p = 0.0066) with sCD14 concentrations higher in febrile episodes (3.03×10^6^ pg/mL [IQR = 2.17×10^6^ pg/mL –3.89×10^6^ pg/mL]) compared with afebrile episodes (2.51×10^6^ pg/mL [IQR = 1.86×10^6^ pg/mL–3.08×10^6^ pg/mL]) (Figure 3a). Stratification of the febrile episodes into those with bacterial findings, viral findings or FUO showed no significant correlation (p = 0.0740) (Figure 3b). Upon further classification of episodes with bacterial findings into Gram-positive bacteraemia and Gram-negative bacteraemia, a difference (p = 0.030) was observed (Figure 3c). sCD14 concentration in FUO episodes (3.20×10^6^ pg/mL [IQR = 2.29×10^6^ pg/mL–3.80×10^6^ pg/mL]) were similar to those with Gram-negative bacteraemia (3.34×10^6^ pg/mL [IQR = 2.52×10^6^ pg/mL–4.01×10^6^ pg/mL]) whilst Gram-positive bacteria findings were observed with a lower median sCD14 concentration of 2.55×10^6^ pg/mL (IQR = 1.87×10^6^ pg/mL–3.07×10^6^ pg/mL). No correlation was observed between viral findings and sCD14 concentrations. Association between sCD14 concentration and underlying disease was not observed.

The endotoxin and sCD14 concentrations were not intercorrelated in this cohort. Similar results were obtained when analyses were limited only to febrile episodes.

## Discussion

In this cohort of iatrogenic neutropaenic adults, we found elevated plasma levels of endotoxin and sCD14 in febrile neutropaenic episodes where no other microbiological findings have been documented, implicating microbial translocation as a potential contributor towards their febrile status. We further observed a correlation between the use of prophylactic antibiotics and increased plasma endotoxin concentrations. Previous studies attempting to determine relation of microbial translocation to febrile episodes in a similar patient cohort had presented mixed results. This stem primarily from differences in endotoxin detection methods [13], [15], degree of immunosuppression in patients [12] and small sample sizes. It should also be noted that these studies limited their work to only systemic concentrations of endotoxins [12], [13], [14] or positive faecal bacterial cultures as indicators of microbial translocation. [15] In only one of these studies were pro-inflammatory cytokines measured as well [12]. However, the patients were less immunosuppressed compared to the current study, leading to difficulties for adequate comparison. To this end, effort had been placed to amass a larger study cohort with strict adherence to the inclusion criteria and, endotoxin concentrations measured with established and commonly used commercial assays. Adding to the field, we have, in our study, in addition to measuring plasma endotoxin concentrations, also measured the host receptor for LPS, sCD14 in an attempt to determine the contribution of microbial translocation to fever as a clinical outcome.

With this adult iatrogenic neutropaenic cohort, no apparent correlation between fever and endotoxin concentrations was observed. The median endotoxin concentrations were however, elevated in the whole cohort compared to healthy controls in earlier reports. [11] The corresponding association of sCD14 concentrations to febrile status then suggests that a host response mediated through sCD14 binding of LPS has been initiated, leading to the development of fever as a symptom, implicating afebrile patients potentially as inadequate responders. Admittedly, microbiological findings should be considered as the most likely grounds of fevers and further stratification was performed to tease apart this confounder.

Endotoxin concentrations in FUO episodes were significantly elevated compared to episodes with bacteria findings. While one might expect that presence of bacteria findings in blood should correlate with elevated endotoxin concentrations, it is pertinent to bear in mind that the detection target of the endotoxin assay is the immunoreactive lipid A portion of LPS. [25] The lower endotoxin concentrations observed in episodes with bacterial findings, as well as the similarities in endotoxin concentrations between episodes with bacterial and viral findings, could reflect a situation of availability. An anchor of the LPS molecule to the bacterial outer membrane, exposure occurs only if they are shed or, upon disruption of the bacterial cell wall. Thus, in line with previous studies [26], it should not be surprising that bacteria findings do not necessarily correlate with increase in measurable endotoxins. The lack of accuracy and precision undoubtedly limits the use of the LAL assay in routine clinical practice but it has been the most widely used standardised method for the measure of endotoxin concentration in recent years. The tendency of hydrophobic regions of lipid A to aggregate or binding to the LPS binding protein, could also potentially limit detection.

A corresponding trend was however, not observed with sCD14 concentrations; there were no differences when episodes were stratified based on microbiological findings or lack thereof, with respect to the FUO group. sCD14 concentrations in episodes with Gram negative bacteria findings were higher than those with Gram positive findings, indicating differential responses to bacteria sub-groups as expected. Further, sCD14 concentrations observed in our study were similar to levels found in plasma of HIV-1 infected patients where microbial translocation had been documented. [11], [27], [28].

Unlike endotoxins, sCD14 concentrations in FUO episodes were not higher than in episodes with Gram negative bacteria. sCD14 performs dual roles; in low levels transfers LPS to mCD14 and TLR4 to initiate pro-inflammatory response however at high systemic concentrations, sCD14 act as an attenuator to cell responses by shuttling LPS to lipoproteins. [29] With the elevated endotoxins observed in this cohort, our findings contend that sCD14 could be acting in an inhibitory way to limit excessive bystander damage from prolonged systemic pro-inflammatory responses. Thus, despite higher endotoxin concentrations in the FUO group, there is no corresponding increase in sCD14. The inherent immunosuppressed state of this cohort could also be implicated in the saturation of sCD14 response observed. Monocytes and to a lesser extent, neutrophils have been suggested to be the main sources of sCD14, either by shedding of mCD14 or secreted directly. Antineoplastic treatment for the underlying disease leaves these patients with reduced monocytes and neutrophils hence a limit on sCD14 production is not entirely surprising. A dampened sCD14 secretion could also explain the lack of correlation between endotoxin and sCD14 concentration in this study. Overall, our findings show evidence of microbial translocation, particularly in the FUO episodes through elevated endotoxin and sCD14 concentrations.

Another interesting finding in this study was that the use of prophylactic antibiotics was associated with higher plasma endotoxin concentrations. Liberal use of prophylactic antibiotics is commonplace in iatrogenic neutropaenic patients as a pre-emptive measure to bacterial infections in this highly susceptible patient group. Yet, the extensive use of oral prophylactic antibiotics might predispose a patient to increased risk of microbial translocation with the subsequent presentation of fever and delays in chemotherapy cycles for their underlying haematological malignancy. One could speculate that the use of prophylactic antibiotics had led to excessive remnant bacterial products or altered the bowel microflora, promoting the overgrowth of resistant variants [30], [31]. Correlation of endotoxin concentrations and duration of prophylactic antibiotic use would have been ideal but we were unfortunately not able to accurately determine this parameter in our study.

Collectively, our findings suggest that in these patients, translocation of bacterial products, rather than whole bacteria, from the intestines has occurred. Correlation of our findings with enterocyte function and integrity could provide more insight into the possible mechanisms of translocation and should be considered for future studies.

Arguably, bacterial findings based solely on positive bacterial cultures could lack sensitivity and be an underestimation of episodes with blood-borne bacterial findings. Broad spectrum PCR targeting conserved bacterial genes like the 16 s rDNA could improve bacterial diagnostics once the inherent difficulties with cross-contamination and false positivity have been resolved. [32] Regardless, PCR diagnostics is a viable option to circumvent this limitation and should be considered for future studies. In this study, we have chosen to follow conventional clinical bacteriological routines in order to obtain the current clinical picture. Given the transient nature of circulating endotoxins and sCD14, care had been taken to ensure samples were obtained within a 72 hr window within which, no sampling bias was observed. Additional information with regards to mucositis grading was unfortunately unavailable in this study and should be considered for inclusion in future studies as a measure of mucosal damage. Further, it would have been desirable to perform longitudinal studies in order to determine if there were correlations between endotoxin concentrations and progression to severe infection or, even septic conditions. Unfortunately, this was not possible with the current study but should be considered in other studies with the same patient cohort. In this cross-sectional study, our findings suggest that in the absence of microbiological findings, microbial translocation could contribute to febrile episodes in an adult neutropaenic cohort. This not only adds to the repertoire of potential causes of fever, it also indicates the urgency for suitable biomarkers to distinguish between drug and tumour related fevers (DTRF) and fevers of infectious causes. Further, an association between prophylactic antibiotic use and increased plasma endotoxin concentrations was observed.
